# 3Rs toxicity testing and disease modeling projects in the European Horizon 2020 research and innovation program

**DOI:** 10.17179/excli2020-1463

**Published:** 2020-06-09

**Authors:** Mathieu Vinken

**Affiliations:** 1 Department of In Vitro Toxicology and Dermato-Cosmetology, Vrije Universiteit Brussel, Laarbeeklaan 103, 1090 Brussels, Belgium

**Keywords:** 3Rs, animal-free, in vitro, in silico, Europe, Horizon 2020

## Abstract

The 3Rs concept, calling for replacement, reduction and refinement of animal experimentation, is receiving increasing attention around the world, and has found its way to legislation, in particular in the European Union. This is aligned by growing efforts of the European Commission to support development and implementation of 3Rs methods. The present paper gives an overview of European 3Rs initiatives as part of the different pillars of the Horizon 2020 framework program for research and innovation. Focus is hereby put on projects that address the 3Rs concept in the context of toxicity testing, chemical risk assessment and disease modeling.

## Setting the Scene for 3Rs Research and Innovation in the European Union

Driven by ethical and scientific constraints that emerged a number of decades ago already, there is a clear tendency worldwide to increasingly employ animal-free methods, in particular for toxicological and safety evaluation of chemicals. This mindset has been majorly inspired by the seminal book entitled “The principles of humane experimental technique” published by William Russell and Rex Burch in 1959, introducing the 3Rs, calling for replacement, reduction and refinement of animal testing (Russell and Burch, 1959[6]). The 3Rs concept served as a basis for the European legislation on the protection of animals for experimental and other scientific purposes, first introduced in 1986 as laid down in Directive 86/609/EEC (EEC, 1986[1]), and revised a decade ago in Directive 2010/63/EU (EU, 2010[2]). This has been reinforced in the past few years by a number of more explicit European legislative changes, in particular in the cosmetics field, where animal testing and marketing bans have been imposed (EU, 2009[3]). An increasing number of countries worldwide have followed the ban on animal testing for cosmetics. In fact, Europe is a world pioneer regarding animal welfare and spearheads the global 3Rs alternative methods field. The European Commission follows a number of strategies in order to do so. In this respect, the European Union Reference Laboratory for Alternatives to Animal Testing (EURL ECVAM), mandated under Directive 2010/63/EU, is not only responsible for validation of 3Rs alternative methods, but also for promoting their regulatory acceptance and disseminating information regarding 3Rs alternative methods. Through EURL ECVAM, the European Commission plays an active role at the level of the Organization for Economic Co-operation and Development (OECD) in the regulatory acceptance of 3Rs alternative methods as well as their international adoption (https://ec.europa.eu/jrc/en/eurl/ecvam). Furthermore, the European Commission teamed up with 8 European trade associations and 36 individual companies from relevant business sectors in a unique public-private partnership called the European Partnership for Alternative Approaches to Animal Testing (EPAA). EPAA is committed to pool knowledge and resources in order to accelerate the development, validation and acceptance of 3Rs alternative approaches for regulatory testing (https://ec.europa.eu/growth/sectors/chemicals/epaa_en). The European Commission also strongly supports research and development of 3Rs alternative methods as part of its framework program for research and innovation called Horizon 2020 (H2020). The H2020 program, which is the successor of the 7^th^ framework program (FP7), has a budget of 80 billion Euro available spread over 7 years *i.e.* from 2014 to 2020. The core of the H2020 program relies on 3 pillars, namely excellent science (pillar 1), industrial leadership (pillar 2) and societal challenges (pillar 3), each that consist of specific funding programs (Table 1(Tab. 1)) (https://ec.europa.eu/programmes/horizon2020/en). 

The present paper gives an overview of relevant completed and ongoing research projects in the H2020 program focused on the development of 3Rs alternative methods. The major resource consulted in order to do so was the Community Research and Development Information Service (CORDIS) (https://cordis.europa.eu/), which is the primary public repository and portal of the European Commission to disseminate information on all funded European research projects.

## 3Rs Projects in H2020 Pillar 1 (Excellent Science)

### VESCEL: vascular engineering-on-chip using differentiated stem cells

The VESCEL project (2015-2020; https://www.utwente.nl/en/eemcs/bios/research/) was funded by the European Research Council and has developed a technology to enable the use of differentiated human induced pluripotent stem cells to engineer blood vessels-on-chip that constitute disease models for thrombosis and neurodegenerative diseases. Such models serve translational research purposes and replace animal testing.

### CryoProtect: a new cryoprotectant formulation for the next generation of high-throughput screening toxicology tests

The CryoProtect project (2016-2017; https://environment.leeds.ac.uk/see-research/dir-record/research-projects/788/cryoprotect-erc-proof-of-concept) was funded by the European Research Council and developed a strategy for cryopreservation of cells within multiwell plates, allowing them be shipped to laboratories around the world and thawed only when they are needed. This helps to optimize *in vitro* testing, which in turn reduces animal experimentation.

### OCLD: tracking the dynamics of human metabolism using spectroscopy-integrated liver-on-chip microdevices

The OCLD project (2016-2021; https://www.nahmias-lab.com/) is funded by the European Research Council and generates a liver-on-chip device capable of tracking the metabolism of tissue engineered livers in real time, enabling an accurate assessment of chronic liver toxicity and the deconstruction of complex metabolic regulation during nutritional events. OCLD will replace animal studies in toxicity testing.

### Mini Brains: cerebral organoids: human mini brains in a dish open up new possibilities for drug development in neurodegenerative and developmental diseases

The Mini Brains project (2017-2018; https://www.imba.oeaw.ac.at/) was funded by the European Research Council and yielded a human pluripotent stem cell-derived 3D organoid culture system. This cerebral organoid system can be used for investigating brain disorders and testing new drugs, thereby offering a 3Rs alternative to animal experiments.

### TOXANOID: pharmacological safety testing in human adult stem cell-derived organoids

The TOXANOID project (2017-2018; https://www.fabiodisconzi.com/open-h2020/projects/209165/index.html) was funded by the European Research Council and developed a 3D culture system that allows to expand human tissue stem cells from intestine and liver as organoids and differentiate them to the respective functional adult tissue *in vitro*. Besides alleviating costs, TOXANOID helped to overcome practical and ethical pitfalls in animal-based testing.

### NeMatrix: nematode-based screening technology for next-generation drug discovery

The NeMatrix project (2017-2018; https://www.epfl.ch/research/) was funded by the European Research Council and developed a microfluidic platform for fully automated culture and analysis of the roundworm *Caenorhabditis elegans* tailored for drug screening applications. NeMatrix has set an example for alternative model organisms for 3Rs testing in biomedical research and pharmaceutical industries.

### INSITE: development and use of an integrated in silico-in vitro mesofluidics system for tissue engineering

The INSITE project (2018-2023; https://www.kuleuven.be/english/research/) is funded by the European Research Council and establishes a new mesofluidics set-up for *in vitro* testing of tissue engineering constructs through development of multiscale and multiphysics models that aggregate the available data and use these to design complex constructs and proper mesofluidics settings for *in vitro* testing. This system will allow for a maximum of relevant *in vitro* research *prior* to the *in vivo* phase, thus complying with the 3Rs concept.

### Design2Flow: disposable well-plate inserts and perfusion chambers for easy-to-use and generic microchannel creation in 3D tissue culture

The Design2Flow project (2020-2021; https://www.ukw.de/startseite/) is funded by the European Research Council and generates perfusable 3D cell culture products for pharmaceutical industry. This allows to mimic *in vivo* blood flow and hence provides a suitable *in vitro* alternative for animal experimentation.

### ORCHID: organ-on-chip in development

The ORCHID project (2017-2019; https://h2020-orchid.eu/) was funded by the Future and Emerging Technologies program and created a roadmap for organ-on-chip technology and the framework to build a network of relevant stakeholders. In this way, ORCHID has facilitated drug development, assisted in developing personalized medicine and contributed to reducing animal experiments.

### TISuMR: integrated tissue slice culture and nuclear magnetic resonance metabolomics: a novel approach towards systemic understanding of liver function and disease 

The TISuMR project (2017-2020; http://tisumr.soton.ac.uk/) was funded by the Future and Emerging Technologies program and produced new technology that combines microfluidic lab-on-chip tissue culture with advanced nuclear magnetic resonance spectroscopy. TISuMR focused on liver tissue culture and drug-induced cholestatic liver injury. By doing so, TISuMR not only increased the efficiency and specificity of drug safety testing, but also provided a 3Rs alternative to animal testing.

### MIMIC: mimicking organs-on-chips for high-throughput drug screening and basic research

The MIMIC project (2016-2019; https://www.sheffield.ac.uk/itn-mimic#gsc.tab=0) was funded by the Marie Skłodowska-Curie Actions program and has contributed to organs-on-chip technology by combining cell biology with microfluidics and chip-based techniques for high-throughput drug screening and basic research. MIMIC combined research activities with 3Rs training of early-stage researchers.

### In3: an integrated interdisciplinary approach to animal-free chemical and nanomaterial safety assessment

The In3 project (2017-2020; https://www.estiv.org/in3/) was funded by the Marie Skłodowska-Curie Actions program and aimed at the synergistic development and application of *in vitro* and *in silico* tools for human chemical and nanomaterial safety assessment. In3 focused on human induced pluripotent stem cell-derived tissues and utilized mechanistic toxicology, quantitative adverse outcome pathways, biokinetics, chemo-informatics and modeling approaches to derive testable prediction models. While having a strong research core, In3 mainly aspired training of young researchers in the 3Rs field.

### ChromaFish: chromatographic micro-column development for pharmaceutical applications in zebraFish

The ChromaFish project (2018-2022; https://www.kuleuven.be/english/research/) is funded by the Marie Skłodowska-Curie Actions program and aims at optimizing and applying methods to measure whole-body uptake of drugs in zebrafish, in particular in brain. ChromaFish is another example of alternative model organisms for 3Rs testing in biomedical research and pharmaceutical industries.

### uKNEEversal: a miniaturized 3D in vitro model of human joint to gain new knowledge on osteoarthritis pathophysiology

The uKNEEversal project (2019-2021; https://www.polimi.it/en/) is funded by the Marie Skłodowska-Curie Actions program and aims to generate a comprehensive *in vitro* human model of osteoarthritis to fill gaps of existing preclinical tools as well as to function as a tool for mechanistic and translational research. Furthermore, uKNEEversal provides a solution for ethical issues regarding animal experimentation by providing a 3Rs replacement alternative.

### ChOLLATERAL: generation of an adverse outcome pathway network on cholestatic liver injury for mechanism-based in vitro testing of chemicals

The ChOLLATERAL project (2019-2021; https://www.vub.be/en/research#home) is funded by the Marie Skłodowska-Curie Actions program and aims at elucidating the mechanisms of cholestatic liver injury. This knowledge is used for setting up advanced human-based *in vitro* testing batteries, which help to better predict liver insults induced by chemicals and that avoid the use of animals.

### PTOoC: plug-n-play tool-kit of organ-on-chips

The PTOoC project (2019-2021; https://www.elveflow.com/) is funded by the Marie Skłodowska-Curie Actions program and aims at building modular organ model systems. PTOoC establishes a library of components to produce tissue testing systems in a plug-and-play fashion. This will produce a tool for testing potential therapies in physiologically relevant systems and will thus minimize animal use.

### SiGNATURE: selection of human iPSC-derived cardiomyocytes by single cell gene expression and patch clamp for a mature cardiac model

The SiGNATURE project (2020-2022; https://www.lumc.nl/research/) is funded by the Marie Skłodowska-Curie Actions program and provides a functionally relevant gene signature of human induced pluripotent stem cell-derived cardiomyocytes of use for the modeling of cardiomyocyte autonomous cardiac diseases for personalized drug screening. SiGNATURE will enhance rates of drug discovery and safety, and, at least in part, will replace the use of animal models.

### OpenRiskNet: open e-infrastructure to support data sharing, knowledge integration and in silico analysis and modeling in risk assessment

The OpenRiskNet project (2016-2019; https://openrisknet.org/) was funded by the Research Infrastructures program and has developed an open e-infrastructure providing resources and services to a variety of communities requiring risk assessment, including chemicals, cosmetic ingredients, therapeutic agents and nanomaterials. A number of case studies demonstrated the applicability of the infrastructure in supporting research and innovation in safer product design and risk assessment, and concomitantly, in replacing the use of animal testing in toxicology.

## 3Rs Projects in H2020 Pillar 2 (Industrial Leadership)

### HISENTS: high-level integrated sensor for nanotoxicity screening

The HISENTS project (2016-2019; https://hisents.eu/) was funded by the Leadership in Enabling and Industrial Technologies program, and delivered an advanced nanosafety platform capable of providing high-throughput toxicity screening for the risk assessment of novel nanomaterials. This screening tool enhanced efficiency of hazard profiling of nanomaterials and reduced the use of animals for safety testing.

### DifMATRIX: ground breaking 3D cell culture platform to eliminate animal testing in pharmaceuticals

The DifMATRIX project (2018; https://inocure.cz/) was funded by the Innovation in Small-sized and Medium-sized Enterprises program and has produced a 3D scaffolding platform with drug delivery embedded system for cell culture, which simulates the *in vivo* tissue phenotype *in vitro*. While improving preclinical-to-clinical translation efficiency, DifMATRIX also provided a 3Rs alternative to animal testing models. 

### HypoSkin: unique breakthrough ex vivo human skin model to predict efficacy and toxicity of subcutaneous drugs

The HypoSkin project (2018; https://www.genoskin.com/) was funded by the Innovation in Small-sized and Medium-sized Enterprises program and has generated an *in vitro* testing kit containing living *ex vivo* human skin biopsies with subcutaneous fat. HypoSkin enabled to reliably predict the local human's response to the injection of drugs and replaced animal testing.

### mP-CSE: microphysiological circadian platform for safety and efficacy assessment of drugs and cosmetics

The mP-CSE project (2018; https://www.tissuedynamics.com/) was funded by the Innovation in Small-sized and Medium-sized Enterprises program and has generated a multi organ-on-chip platform that uses real-time sensing of tissue function, including mimicking of circadian rhythms, to detect the effect of drug-induced physiological stress in multiple 3D vascular organs. This device reduced cost of drug development, but also established a 3Rs alternative for safety testing.

### QSAREACH: QSAR computational models' self-using platform for EC Regulation REACH

The QSAREACH project (2018-2019; https://protoqsar.com/en/european-comission-sme-instrument/) was funded by the Innovation in Small-sized and Medium-sized Enterprises program and generated an *in silico* platform consisting of quantitative structure-activity relationship models for (eco)toxicological investigation of chemicals, including nanomaterials, in line with European legislation. This forms a full 3Rs replacement alternative for animal testing.

### MOOD: feasibility study for the introduction into preclinical study domain of the first-of-a-kind multi-organ-on-device technology

The MOOD project (2018-2019; https://www.react4life.com/) was funded by the Innovation in Small-sized and Medium-sized Enterprises program and generated a multi-organ-on-device, being an advanced fluidic multichamber bioreactor and a 3D model of breast cancer tissue cultured onto a membrane mimicking the blood vessel barrier. This device reduced time, costs and use of animals in drug development.

### uHeart: a beating heart-on-chip for preclinical early detection of drugs cardiac safety

The uHeart project (2019-2020; http://www.biomimx.com/) was funded by the Innovation in Small-sized and Medium-sized Enterprises program and has produced a beating heart-on-chip by integrating a 3D cell culture and mechanical stimulation. While providing a tool for rapid screening in early drug development, uHeart also provided a 3Rs alternative to animal testing models. 

### OrganoPlate Graft: organ-on-a-chip technology for in vitro grafting and vascularization of 3D tissues

The OrganoPlate Graft project (2019-2021; https://mimetas.com/) is funded by the Innovation in Small-sized and Medium-sized Enterprises program and will deliver a high-throughput *in vitro* culture method for vascularized tissue. OrganPlate Graft will enable design of novel types of experiments in research, development and clinical settings, and will replace animal tests.

### Droplet Microarray: miniaturized system for screening of primary cells based on droplet microarray

The Droplet Microarray project (2020-2022; http://www.aquarray.com/) is funded by the Innovation in Small-sized and Medium-sized Enterprises program and focuses on the miniaturization of microplates using primary cells for drug discovery purposes. This will enable personalized medicine and will reduce use of animals in pharmaceutical industry.

## 3Rs Projects in H2020 Pillar 3 (Societal Challenges)

### EuroMix: a tiered strategy for risk assessment of mixtures of multiple chemicals

The EuroMix project (2015-2019; https://www.euromixproject.eu/) was funded under the Food topic and developed a tiered strategy for the risk assessment of mixtures of multiple chemicals derived from several sources across different life stages. EuroMix abundantly used *in vitro* assays and *in silico* tools, and hence led to a reduction of the use of animals for safety testing of chemicals.

### EDC-MixRisk: integrating epidemiology and experimental biology to improve risk assessment of exposure to mixtures of endocrine disruptive compounds

The EDC-MixRisk project (2015-2019; https://edcmixrisk.ki.se/) was funded under the Health topic and focused on effects of mixtures of endocrine disrupting chemicals on children. EDC-MixRisk developed an appropriate mixture risk assessment methodology implemented in an interoperational data and model platform and relied on animal-free research.

### EU-ToxRisk: an integrated European flagship program driving mechanism-based toxicity testing and risk assessment for the 21^st^ century

The EU-ToxRisk project (2016-2021; http://www.eu-toxrisk.eu/) is funded under the Health topic and establishes a mechanism-based testing strategy for animal-free chemical safety assessment of chemicals of diverse sectors. It is considered the flagship 3Rs project in Europe, as it integrates all concepts of 21^st^ century toxicity testing, including human-based testing devoid of animals by fully relying on *in vitro* and *in silico* experimentation.

### InSilc: in silico trials for drug-eluting BVS design, development and evaluation 

The InSilc project (2017-2020; https://insilc.eu/) was funded under the Health topic and aimed at delivering an *in silico* clinical trial platform for designing, developing and assessing drug-eluting bioresorbable vascular scaffolds by building on the comprehensive biological and biomedical knowledge and advanced modeling approaches to simulate their implantation performance in individual cardiovascular physiology. InSilc contributed to reduction of development costs and shorting of time-to-market, and the implementation of the 3Rs.

### HBM4EU: European human biomonitoring initiative

The HBM4EU project (2017-2021; https://www.hbm4eu.eu/) is funded under the Health topic and aims at generating knowledge to inform the safe management of chemicals by using human biomonitoring to understand human exposure to chemicals and resulting health impacts. HBM4EU will adapt chemical risk assessment methodologies to use human biomonitoring data, thereby avoiding reliance on animal experimentation.

### EURION: European cluster to improve identification of endocrine disruptors

EURION (2019-2024; https://eurion-cluster.eu/) is a cluster group of 8 research projects (ATHENA, ERGO, SCREENED, ECDMET, GOLIATH, OBERON, ENDpoiNTS and FREIA) under the Health topic focused on testing and screening methods to identify endocrine disrupting chemicals. Each project in the cluster addresses a different aspect of new testing and screening methods to identify endocrine disrupting chemicals, thereby embracing the 3Rs concept as much as possible. 

## Conclusions and Perspectives

According to the most recent report of the European Commission on the statistics on the number of animals used for experimental and other scientific purposes in the member states of the European Union, 2.18 million animals were used for regulatory testing purposes in 2017, which is roughly one fourth of all animals used during that year. A total of 8 % of all animals was used for toxicological and other safety evaluation purposes. Although this number slightly decreased compared to previous years, this still implies a considerable number of animals (EU, 2020[4]). Intensive efforts worldwide try to reduce and replace the use of animals, in particular for toxicity testing of chemicals. In this respect, the US Environmental Protection Agency (US EPA) issued a *memorandum *in 2019 announcing that it will reduce its requests for and funding of mammal studies by 30 % towards 2025, and eliminate all mammal study requests and funding by 2035. Furthermore, the US EPA stated that the regulatory requirements for risk assessment will be refined and an increased flexibility in risk assessment framework will be implemented to facilitate the incorporation of non-animal methods (US EPA, 2019[7]). In 2016, the Netherlands National Committee for the Protection of Animals used for Scientific Purposes published a strategy to phase out animal experiments by 2025 (NCad, 2016[5]). Although the ambition was later on loosened to making research largely free of animal testing, the Netherlands still aspire to become world leader in innovations without laboratory animals in 2025. At the European level, a multitude of instruments is being used since many years to implement the 3Rs principles, not only in toxicology and risk assessment, but in life science research and education in general. In FP7, the European Commission spent about 200 million Euro on supporting animal-free toxicology projects (https://ec.europa.eu/environment/chemicals/lab_animals/3r/research_en.htm). A considerable part of this budget was dedicated to public-private partnerships, including the Safety Evaluation Ultimately Replacing Animal Testing consortium (SEURAT; http://www.seurat-1.eu/) and the Innovative Medicines Initiative (IMI; https://www.imi.europa.eu/), with the trade association Cosmetics Europe and the European Federation of Pharmaceutical Industries and Associations, respectively. Some of those initiatives, in particular IMI, are continued in the H2020 program, with projects such as eTRANSAFE (https://etransafe.eu/), focused on the use of *in silico* tools in the pharmaceutical sector, and VAC2VAC (http://www.vac2vac.eu/), which addresses *in vitro* method development for vaccine testing. In fact, the 3Rs are fully intertwined in the 3 pillars of the H2020 program. While pillar 1 (excellent science) and pillar 2 (industrial leadership) focus in general on the development of new animal-free methodologies, such as advanced *in vitro* models and *in silico* tools, multidisciplinary and intersectoral projects in pillar 3 (societal challenges) rather address the integration of such new technical know-how in view of setting up human-based strategies and workflows for next generation toxicity testing and risk assessment of chemicals fully devoid animals. Such pillar 3 projects are increasingly being encouraged to team up in clusters in order to maximize cross-fertilization of knowledge and hence to create synergistic outcome. In this context, a new call for pillar 3 projects under the Health topic was published in 2019 (https://ec.europa.eu/info/funding-tenders/opportunities/portal/screen/opportunities/topic-details/sc1-bhc-11-2020) and is expected to result in 3-4 projects that will intensively collaborate under the theme “advancing the safety assessment of chemicals without the use of animal testing” as of 2021. Also in 2019, the European Commission published the proposal for the H2020 follow-up research and innovation program called Horizon Europe for the period 2021-2027 (https://ec.europa.eu/info/horizon-europe-next-research-and-innovation-framework-programme_en). With some changes compared to its H2020 predecessor, the Horizon Europe program has kept a 3-pillar structure and foresees ample opportunities for 3Rs research, innovation, collaboration and education. This will not only consolidate Europe's world-leading 3Rs position, but first and foremost will save the lives of millions of animals, while meeting the ever increasing regulatory human safety requirements of chemicals.

## Funding

No funding was received for this paper.

## Conflict of interest

No conflicts of interest are to report.

## Author’s contributions

Mathieu Vinken is the sole author of this paper.

## Figures and Tables

**Table 1:**
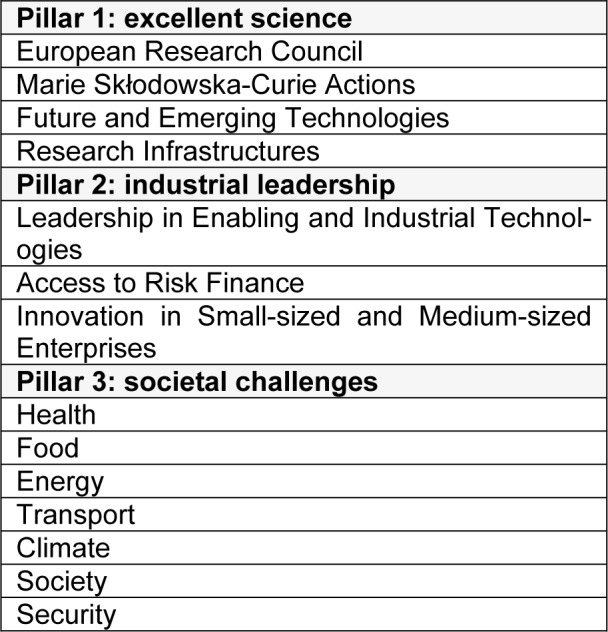
Pillars of the H2020 program (https://ec.europa.eu/programmes/horizon2020/en)
